# A systematic review on the economic burden of interstitial lung disease and the cost-effectiveness of current therapies

**DOI:** 10.1186/s12890-022-01922-2

**Published:** 2022-04-20

**Authors:** Alyson W. Wong, John Koo, Christopher J. Ryerson, Mohsen Sadatsafavi, Wenjia Chen

**Affiliations:** 1 Department of Medicine, University of British Columbia, Vancouver, Canada; 2 Centre for Heart Lung Innovation, St. Paul’s Hospital, Ward 8B - Providence Wing, 1081 Burrard St., Vancouver, V6Z 1Y6 Canada; 3 Respiratory Evaluation Sciences Program, Collaboration for Outcomes Research and Evaluation, Faculty of Pharmaceutical Sciences, University of British Columbia, Vancouver, Canada; 4 Health Systems and Behavioural Sciences, Saw Swee Hock School of Public Health, National University of Singapore, Singapore, Singapore

**Keywords:** Lung diseases, interstitial, Costs and cost analysis

## Abstract

**Background:**

The economic burden of interstitial lung disease (ILD) is unknown, limiting informed resource allocation and planning. We sought to conduct the first systematic review on the direct, indirect, and overall costs associated with ILD and to evaluate the cost-effectiveness of current therapies globally.

**Methods:**

We conducted systematic reviews of ILD disease cost studies and cost-effectiveness analyses (CEAs) using MEDLINE, Embase, and Web of Science databases between 2000 and 2020. We compared ILD costs between countries according to the share of costs towards each country’s respective gross domestic product (GDP) per capita. Costs are reported in 2020 USD.

**Results:**

We identified 25 disease cost studies and 7 CEAs. The direct medical costs ranged between $1824 and $116,927 annually per patient (median $32,834; 14–180% of GDP per capita in Western countries). The leading drivers of direct costs were inpatient (55%), outpatient (22%), and medication costs (18%), based on pooled estimates. Annual indirect costs ranged from $7149 to $10,902 per employed patient (median $9607; 12–23% of GDP per capita). Among the 7 CEAs, only 1 study (14%) showed an ILD therapy (ambulatory oxygen) was cost-effective compared to best supportive care.

**Conclusion:**

The direct and indirect costs associated with ILD are consistently high in all countries with available data, with cost-effectiveness profiles of new therapies generally undesirable. Globally, the median total direct cost for ILD equates to 51% of a country’s GDP per capita and has been increasing over time.

**Supplementary Information:**

The online version contains supplementary material available at 10.1186/s12890-022-01922-2.

## Background

Interstitial lung diseases (ILDs) are a collection of disorders characterized by inflammation and/or fibrosis of the lung parenchyma that can lead to lung function decline, reduced quality of life, and early mortality [1]. ILD is prevalent (256 per 100,000 people) and associated with substantial healthcare costs [2, 3]. Despite its potential impact on health systems, the evidence on the healthcare costs across different forms of ILD is scarce. A comprehensive synthesis of the economic burden of ILD and the value for money of its treatments is crucial for resource allocation and planning.

Economic evaluation can be categorized into partial evaluations that examine the cost or consequences of a single intervention or full economic evaluations that examine the costs and consequences of two or more interventions [4]. Cost-effectiveness analysis (CEA) is a type of full economic evaluation that provides an objective decision-making framework for determining the value-for-money potential of competing interventions [4]. In CEAs, the comparison between interventions is summarized by the incremental cost-effectiveness ratio (ICER), which describes the additional cost per additional unit of health gain for one intervention compared to the other [5]. A common measure of health gain is the quality adjusted life year (QALY), where 1 QALY equals 1 year of full health [6]. The incremental net benefit (INB) is another measure of cost-effectiveness and provides additional information, such as the probability that a new treatment is cost-effective for different willingness-to-pay values [7].

The purpose of this systematic review was to evaluate and synthesize current evidence from around the world and address the following objectives: (1) determine the global direct, indirect, and overall costs associated with ILD and (2) evaluate the cost-effectiveness of current therapies in ILD.

## Methods

### Data sources and searches

The protocol for this systematic review was registered on PROSPERO (CRD42020158417). The search was conducted using Embase (Ovid), MEDLINE (Ovid), and Web of Science databases between January 2000 and October 2020, using ILD-related search terms (e.g., “lung diseases, interstitial” or “pulmonary fibrosis”) and health costs (e.g., “economics” or “health care economics and organizations” or “healthcare costs” or “cost effectiveness analysis” or “cost benefit analysis”). The full search protocol is available in the Additional file 1: Supplement. January 1, 2000 was used as the start date because the first ILD diagnostic guideline (specifically for idiopathic pulmonary fibrosis) was published this year [8]. Classification documents for other ILDs were published afterwards. Thus, limiting studies to those published after 2000 ensured standardized case definitions for ILDs and allowed more accurate comparisons between studies.

### Study selection

Disease cost studies were eligible if they included patients ≥ 18 years of age with a diagnosis of ILD, investigated relevant interventions supported by current clinical guidelines or standard of care, and included at least one cost outcome. Eligible CEA studies included study participants with ILD, compared an intervention supported by current clinical guidelines to another intervention or standard of care, and reported an ICER. Studies that were non-English, not original research, or a case series with < 10 patients were excluded. Studies that did not provide specific ILD-related costs (e.g., costs provided for all patients with sarcoidosis and not specifically for those with ILD) were excluded.

### Data extraction and quality assessment

Screening for eligible studies and data extraction were completed by two reviewers, with discrepancies determined by consensus. References of included citations were screened to identify additional publications that may have been missed during the initial search. Extracted data included study characteristics (e.g., type of economic evaluation, study period, country) and study results (e.g., type of costs and their amounts). The gross domestic product (GDP) per capita for the study period were also obtained based on the study country [9]. The Consolidated Health Economic Evaluation Reporting Standards (CHEERS) was used to evaluate the quality of reporting [10]. Components of the CHEERS criteria that were applicable to disease cost studies were used to evaluate their risk of bias.

### Statistical methods

Costs are reported as mean annual cost per patient, unless otherwise specified. For studies that reported mean annual costs over multiple years, we calculated the annual average to maximize data utilization. For studies that used multiple diagnostic criteria to identify patients with ILD, we used the most specific criteria to increase the likelihood that patients with ILD were being studied. Given costs differ before and after ILD diagnosis, we included costs after the diagnosis of ILD to allow comparison between included studies [11]. To make comparisons meaningful, we compared ILD costs between countries according to the share of costs towards their GDP per capita. The GDP per capita during the study period was used and reported in 2020 USD [9, 12].

The ICER was calculated by dividing the differences in cost by the differences in QALY of two treatment strategies. For the INB, the monetary value of benefit for an intervention was first determined by multiplying a willingness to pay value (the standard US threshold of $50,000 was used) by the incremental units of effectiveness (e.g., QALYs) [13]. The incremental cost of the intervention was then subtracted from this amount. Given the commonly used $50,000 threshold is arbitrary, a sensitivity analysis using different thresholds (1-time and 3-times the country’s GDP) was conducted to evaluate whether treatments remained cost-effective at different thresholds that were more reflective of a country’s willingness-to-pay. A positive INB reflects a cost-effective intervention. If there were multiple CEAs that evaluated the same intervention, we reported the median and range for the ICER and INB.

All costs were converted to 2020 USD using currency exchange rates and were adjusted for inflation using the Consumer Price Index [14]. Costs represent attributable costs, rather than excess costs relative to a control. Statistical analyses were conducted using R version 4.0.3.

## Results

### Literature search

**Fig. 1:**
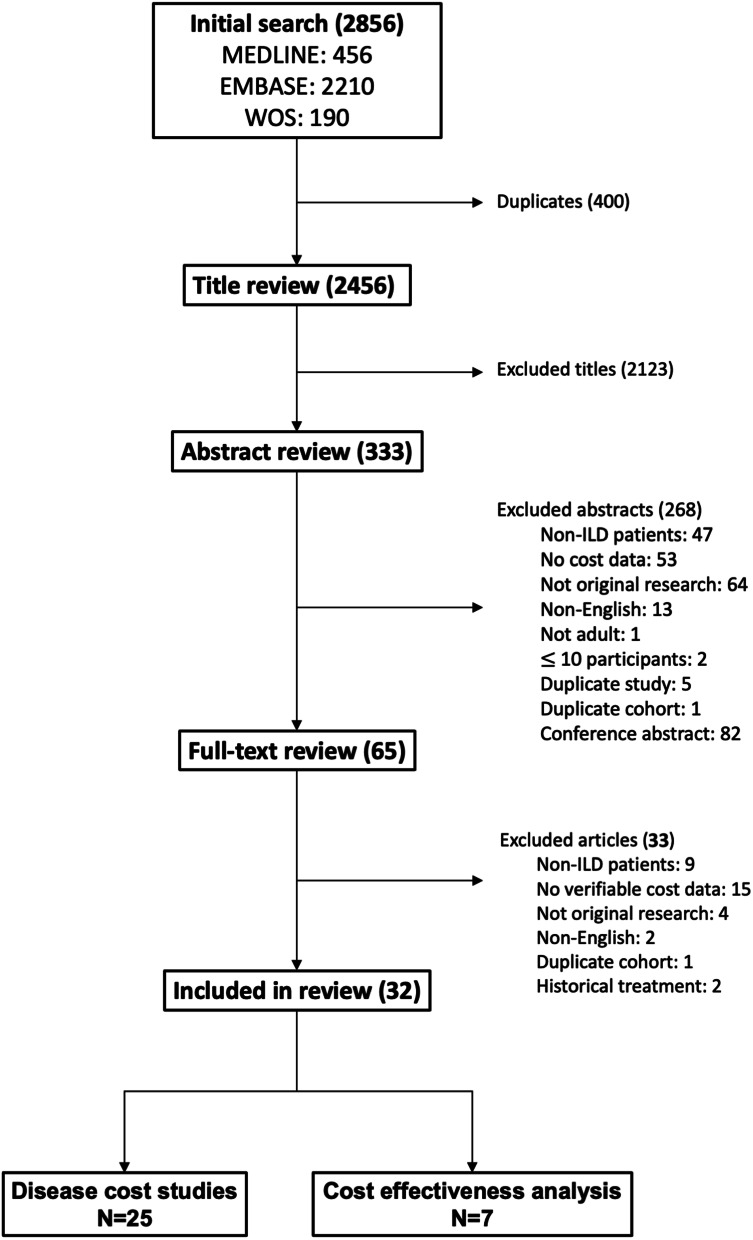
Systematic review flow diagram. The final studies included 25 disease cost studies (evaluate direct and/or indirect costs associated with ILD) and 7 cost effectiveness analyses (compare costs and outcomes of 2 or more interventions). *Abbreviations:* WOS, Web of Science

The initial literature search yielded 2856 citations with 2456 unique citations after removing duplicates (Fig. 1). Titles and abstracts were screened, leaving 65 full texts to review, of which 32 met eligibility criteria and were included in data extraction. There were 25 disease cost studies (measuring the direct and/or indirect costs associated with ILD) and 7 CEAs (comparing health outcomes and costs between 2 or more interventions in order to determine the cost required to gain one unit of health outcome) [15]. Cost utility analyses where the health utility is specifically used as the measure of health gain were included under CEAs. Other forms of economic evaluation (e.g., cost minimization and cost benefit analyses) were not identified in the search.

### Characteristics and findings of included studies

There were 21 studies that reported only direct costs (14 IPF, 4 connective tissue disease-associated ILD [CTD-ILD], 2 fibrotic ILD, and 1 idiopathic interstitial pneumonia), 2 studies that reported only indirect costs (1 fibrotic ILD and 1 CTD-ILD), and 2 studies that reported both direct and indirect costs (1 IPF and 1 CTD-ILD). Four of the 6 CTD-ILD studies specifically focused on systemic sclerosis. Most of the disease cost studies were based on North American populations (12 United States and 4 Canada), with the remaining studies from European countries (7), Korea (1), and Australia (1). There were 7 CEAs (5 evaluated antifibrotics, 1 ambulatory oxygen, and 1 lung transplantation), of which 6 were conducted in patients with IPF. All CEAs were based in European countries.

#### Direct costs

**Fig. 2:**
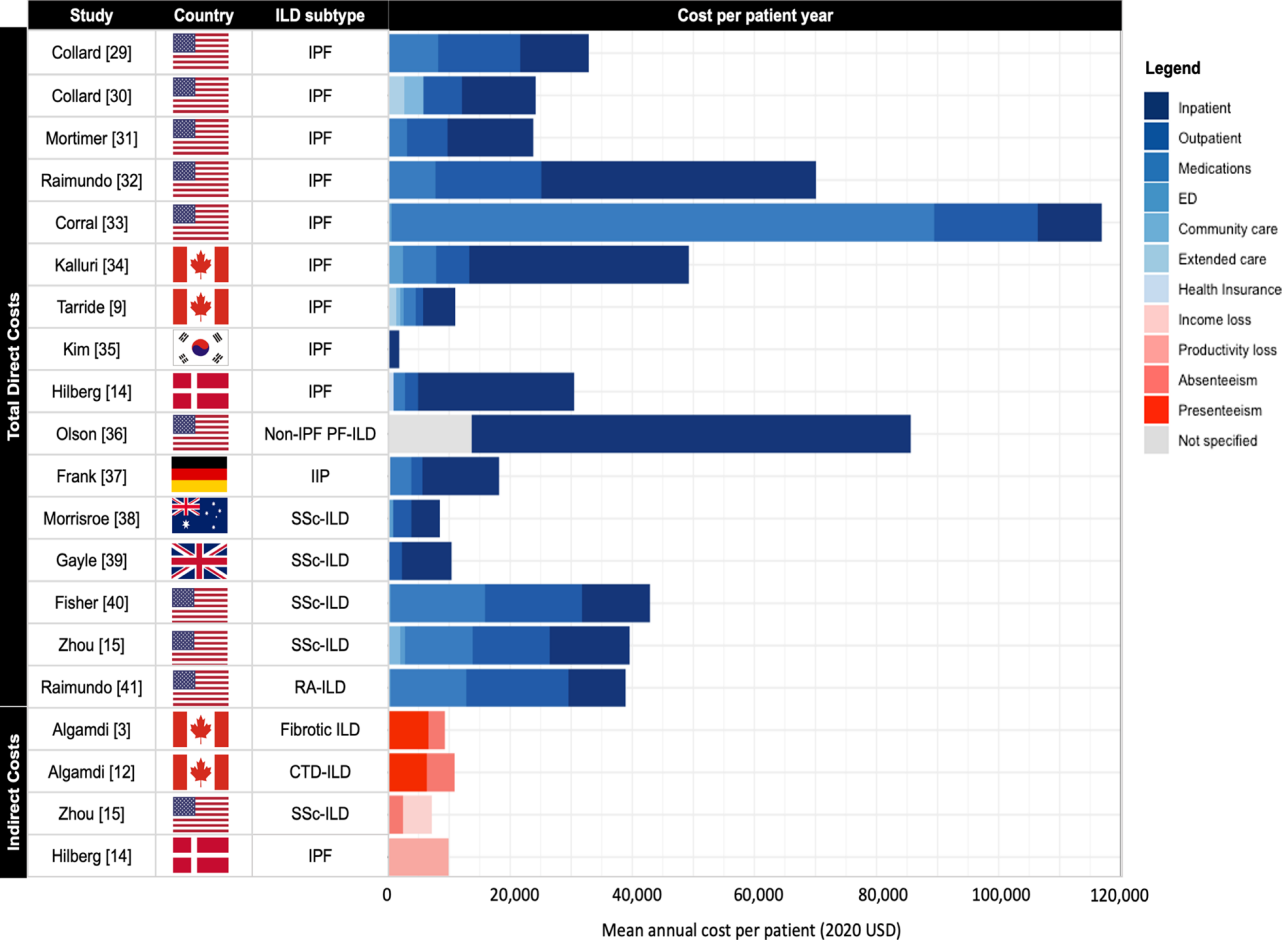
Disease cost studies for patients with ILD. The blue and red bars represent different cost components for total direct and indirect costs, respectively. Inpatient costs include emergency department visits. Outpatient costs include medical services such as physician visits, laboratory tests, procedures, and health insurance. Community care refers to home support services, while extended care refers to long term care and hospice. *Abbreviations:* CTD, connective tissue disease; IIP, idiopathic interstitial pneumonia; IPF, idiopathic pulmonary fibrosis; PF-ILD, progressive fibrosing interstitial lung disease; SSc, systemic sclerosis; RA, rheumatoid arthritis

The total direct costs ranged from $1824 to $116,927 annually per patient (median $32,834) (Table 1). The leading drivers of costs were inpatient (55%), outpatient (22%), and medication costs (18%), based on pooled estimates from studies that provided cost breakdowns (Fig. 2). Most studies were conducted before antifibrotics became part of standard care (in 2014 for most countries). Therefore, the contribution of medications towards ILD costs is likely underrepresented. In patients with IPF, the range of total direct costs increased from $1824 to $70,051 annually per patient before antifibrotics and up to $49,251 to $116,927 afterwards. There were 7 studies that investigated specific types of direct costs (6 hospitalization and 1 cryobiopsy costs) (Table 2). There was a wide range for hospitalization costs, from $5410 to $19,136 annually per patient.

**Table 1:** Total direct and indirect costs of ILD.

Study	Country	Study period	Cost component	Population	Sample size	Mean annual cost per patient (2020 USD)	GDP per capita (2020 USD)	Proportion of GDP per capita (%)
Direct Costs								
Collard [2]	US	2001–2008	Total direct	IPF	9286	32,834	58,160	56
Collard [40]	US	2000–2011	Total direct	IPF	7855	24,198	57,395	42
Mortimer [41]	US	2008–2014	Total direct	IPF	4716	23,773	60,183	40
Raimundo [42]	US	2009–2011	Total direct	IPF	3619	70,051	57,395	122
Corral [33]	US	2014–2018	Total direct	IPF	1455	116,927	64,999	180
Kalluri [34]	Canada	2012–2018	Total direct	IPF	2768	49,251	47,879	103
Tarride [11]	Canada	2006–2011	Total direct	IPF	8683	10,991	60,088	18
Kim [43]	Korea	2009–2013	Total direct	IPF	18,006	1,824	30,200	6
Hilberg [19]	Denmark	2003–2009	Total direct	IPF	120	30,513	70,167	43
Olson [44]	US	2014–2016	Total direct	Non-IPF PF-ILD	373	85,589	62,567	137
Frank [45]	Germany	2010–2013	Total direct	IIP	14,453	18,196	51,437	35
Morrisroe [46]	Australia	2008–2015	Total direct	SSc-ILD	335	8,473	61,975	14
Gayle [47]	England	2005–2016	Total direct	SSc-ILD	127	10,398*	44,264	23
Fisher [48]	US	2003–2014	Total direct	SSc-ILD	219	42,878	60,183	71
Zhou [20]	US	2005–2015	Total direct	SSc-ILD	479	39,560	62,092	64
Raimundo [49]	US	2004–2013	Total direct	RA-ILD	11,845	38,907	59,001	66
Indirect Costs								
Algamdi [16]	Canada	2015–2017	Productivity loss	Fibrotic ILD	148	9,313	47,650	20
Algamdi [17]	Canada	2015–2017	Productivity loss	CTD-ILD	113	10,902	47,650	23
Zhou [20]	US	2005–2015	Productivity loss	SSc-ILD	479	7,149	62,092	12
Hilberg [19]	Denmark	2003–2009	Income loss	IPF	120	9,901	70,167	14

Costs are attributable (i.e., total costs directly associated with the ILD, rather than excess costs relative to a control). The GDP per capita is based on the study country. The proportion of GDP per capita represents the mean annual cost per patient relative to the country’s GDP per capita based on 2020 USD [12]. Productivity loss includes absenteeism and presenteeism. *Median cost. *Abbreviations*: GDP, gross domestic product; IPF, idiopathic pulmonary fibrosis; PF-ILD, progressive fibrosing interstitial lung disease; RA, rheumatoid arthritis; SSc, systemic sclerosis

**Table 2:** Costs for specific direct cost components

Study	Country	Study period	Cost component	Population	Sample size	Mean cost (2020 USD)	GDP per capita (2020 USD)	Proportion of GDP per capita (%)
Yu [50]	US	2006–2011	Hospitalization	IPF	1735	16,205	57,395	28
Mooney [51]	US	2009–2011	Hospitalization	IPF	22,350	19,136	57,395	33
Fan [52]	US	2014–2016	Hospitalization	IPF	300	15,202	62,567	24
Cottin [53]	France	2008–2013	Hospitalization	IPF	6476	5410^†^	47,333	11
Navaratnam [54]	England	1998–2010	Hospitalization	IPF	26,766	2964*	46,927	6
Pedraza-serra [55]	Spain	2004–2013	Hospitalization	IPF	12,739	7886	32,294	24
Hernandez-Gomez [56]	Spain	2011–2014	Cryobiopsy	Fibrotic ILD	33	472	32,252	1

The proportion of GDP per capita represents the mean annual cost per patient relative to the country’s GDP per capita based on 2020 USD [12]. The cost per hospitalization or cryobiopsy procedure is shown. *cost per number of bed days. ^†^Median value of first hospitalization. *Abbreviations*: GDP, gross domestic product; IPF, idiopathic pulmonary fibrosis

**Fig. 3:**
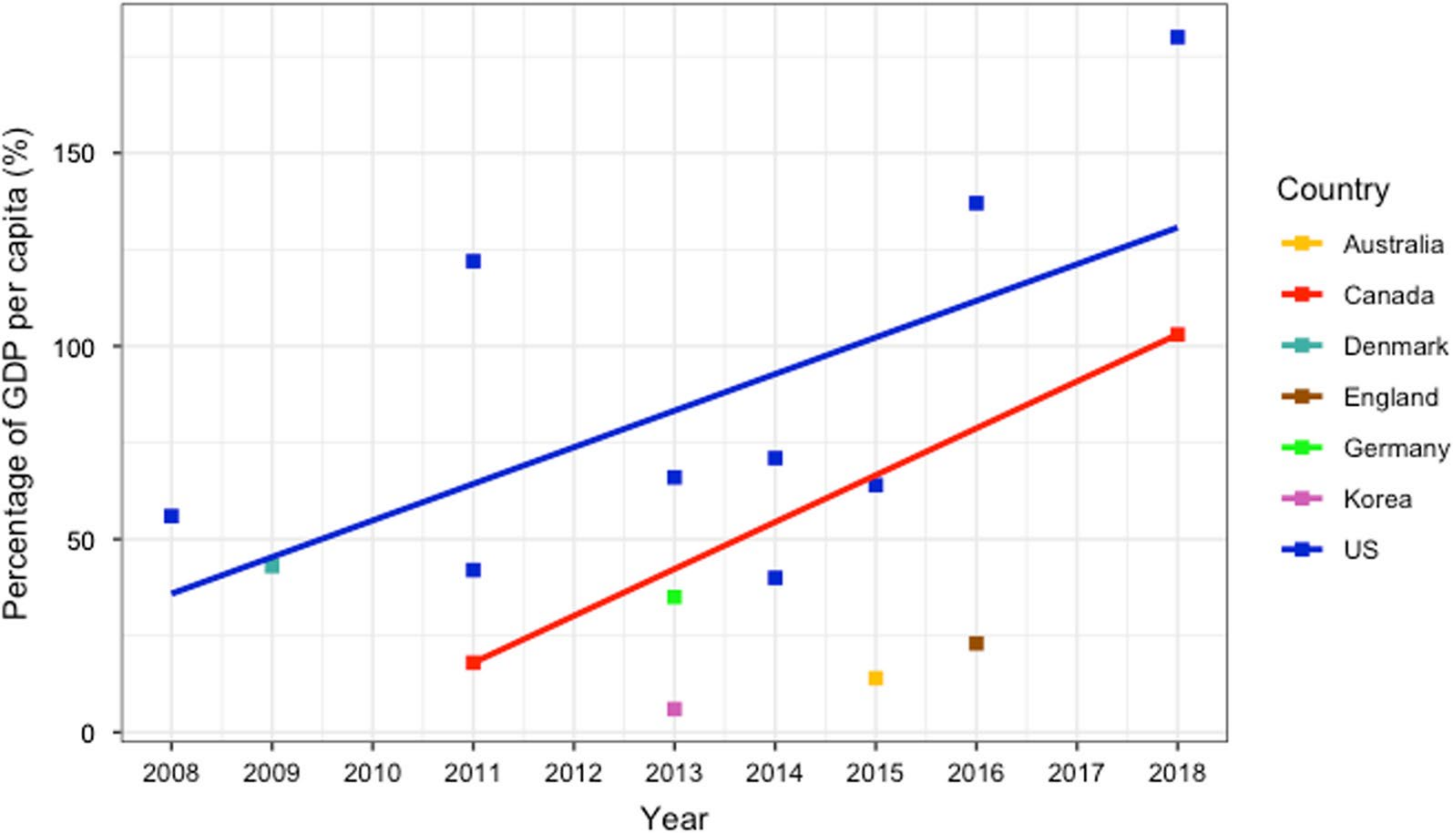
Annual total direct cost per patient as a percentage of GDP per capita. For studies with an annual cost over a range of years, the last year is shown in the graph. A line of best fit is shown for countries that had ≥ 2 studies (US and Canada)

The total direct costs in ILD varied among countries. On average, the median annual cost per patient was 50% of the GDP per capita. There was a stark contrast between spending in Western countries and Korea (the only non-Western country). The US was the highest spending country with the mean annual cost per patient being up to 180% of GDP per capita in one study, while the costs in Korea were 6% of GDP per capita (Fig. 3).

#### Indirect costs

The cost components used to determine indirect costs was variable. Two Canadian studies specifically focused on work productivity loss in fibrotic ILD (Table 1) [16, 17]. Productivity loss associated with absenteeism and presenteeism refers to loss as a result of absence from work and working with limitations due to illness, respectively [18]. Productivity loss (absenteeism and presenteeism) was similar between the two studies with a mean annual cost of $9313 (20% of GDP per capita) and $10,902 per patient (23% of GDP per capita), respectively. In other studies, indirect costs referred to income loss or unemployment benefits and did not include presenteeism [19, 20]. The method of calculating indirect costs also varied. For example, some studies used a validated questionnaire such as the Work Place and Activity Impairment which asks patients to recall how many hours were attributed to absenteeism and presenteeism over the prior 1 week period [16, 17, 21], while other studies calculated days of work loss based on medical and disability claims [19, 20].

#### Cost-effectiveness analysis

Despite a broad search strategy for CEAs in ILD, only 7 studies were identified, of which 5 focused on IPF and antifibrotic therapy. The following models were used in the CEA studies: 5 Markov models, 1 decision tree, and 1 microsimulation (Table 3). The perspective of a CEA refers to the point of view adopted when deciding which costs and health benefits will be included. Three studies used a societal perspective which is the broadest perspective that reflects a full range of costs and includes productivity loss [22–24]. Another 3 studies were from the perspective of the UK National Health Service [25–27], while 1 study was from the specific perspective of the Belgian healthcare payer [28]. All studies used a lifetime time horizon, except for 1 study that evaluated cost-effectiveness of ambulatory oxygen over 2 weeks [27].

**Table 3:** Overview of cost-effectiveness analyses

First author Study year	ILD	Model	Perspective	Time horizon	Treatment (tx)	Tx cost	Tx QALY /person	Control	Control cost	Control QALY /person	ICER	INB
**Pharmacologic therapies**												
Clay [22] 2019	IPF	Markov	Societal	Lifetime	Nintedanib	140,486	4.50	**Pirfenidone**	133,592	5.20	9,849	-41,894
Porte [24] 2018	IPF	Markov	Societal	Lifetime	**Nintedanib**	109,100	3.34	Pirfenidone	118,025	3.29	-178,500	11,425
Rinciog [26] 2017	IPF	Markov	UK NHS & PSS	Lifetime	**Nintedanib**	148,950	3.50	Pirfenidone	152,986	3.45	-80,720	6,536
Loveman [25] 2015	IPF	Markov	UK NHS & PSS	Lifetime	Nintedanib	272,273	4.01	**Pirfenidone**	128,523	3.34	214,552	-110,250
Rinciog [28] 2020	IPF	Markov	Healthcare payer	Lifetime	**Nintedanib**	138,191	3.36	Pirfenidone	151,705	3.28	-168,925	17,514
Clay [22] 2019	IPF	Markov	Societal	Lifetime	Nintedanib	140,486	4.50	**BSC**	19,039	3.80	173,496	-86,447
Porte [24] 2018	IPF	Markov	Societal	Lifetime	Nintedanib	109,100	3.34	**BSC**	22,056	2.98	241,789	-69,044
Rinciog [26] 2017	IPF	Markov	UK NHS & PSS	Lifetime	Nintedanib	148,950	3.50	**BSC**	38,076	3.10	277,185	-90,874
Loveman [25] 2015	IPF	Markov	UK NHS & PSS	Lifetime	Nintedanib	272,273	4.01	**BSC**	6,014	2.98	258,504	-214,759
Clay [22] 2019	IPF	Markov	Societal	Lifetime	Pirfenidone	133,592	5.20	**BSC**	19,039	3.80	81,824	-44,553
Porte [24] 2018	IPF	Markov	Societal	Lifetime	Pirfenidone	118,025	3.29	**BSC**	22,056	2.98	309,577	-80,469
Rinciog [26] 2017	IPF	Markov	UK NHS & PSS	Lifetime	Pirfenidone	152,986	3.45	**BSC**	38,076	3.10	328,314	-97,410
Loveman [25] 2015	IPF	Markov	UK NHS & PSS	Lifetime	Pirfenidone	128,523	3.34	**BSC**	6,014	2.98	340,303	-104,509
**Non-pharmacologic therapies**												
Whitty [27] 2019	IPF	Decision tree	UK NHS	2 weeks	**Ambulatory oxygen**	128*	3.70*	No oxygen	-	-	35	184,872
Groen [23] 2004	Any ILD	MS	Societal	Lifetime	Lung transplantation	138 M	1203	**No lung transplant**	71.25 M	738	143,548	-17 M

The incremental cost effectiveness ratio (ICER) is the cost per additional quality adjusted life year (QALY) for a given treatment. The incremental net benefit (INB) was calculated using a willingness to pay value of $50,000. INB values > 0 represent cost-effective interventions (bold text). Costs are shown in 2020 USD. *Cost and KBILD values for each of the treatment and control groups were not available. The incremental cost and KBILD score are shown, with the ICER representing the cost per unit increase in KBILD. *Abbreviations:* KBILD, King’s Brief Interstitial Lung Disease; M, million; MS, microsimulation; PSS, personal social services; Tx, treatment; UK NHS, United Kingdom National Health Service

The median ICER for either nintedanib or pirfenidone compared to best supportive care (BSC) was $250,146 for nintedanib (range $173,496 to $277,185) and $318,946 (range $81,824 to $340,303) for pirfenidone. In other words, it costs $250,146 per QALY with nintedanib compared to BSC, while pirfenidone costs $318,946 per QALY. Using the INB, only 1 study showed ILD therapies (ambulatory oxygen) to be cost-effective compared to best supportive care. There were 5 other studies that demonstrated cost-effectiveness when comparing antifibrotic medications (nintedanib and pirfenidone) to one another. Three of the 5 studies found that nintedanib was the dominant strategy (reduced costs and improved QALYs) [24, 26, 28]; while the other 2 studies found pirfenidone to be dominant compared to nintedanib [22]. These therapies remained cost-effective when the threshold used to calculate the INB was changed to 1-time and 3-times the GDP per capita (Additional file 1: Table S1).

There were two CEAs that evaluated non-pharmacologic therapies. One CEA assessed ambulatory oxygen and showed an incremental cost of $35 per additional point in the King’s Brief ILD score (a validated tool to assess health-related quality of life in ILD) compared to no ambulatory oxygen use [27, 29]. The other study assessed lung transplantation and reported an ICER of $143,548 per QALY compared to no lung transplantation [23].

### Quality assessment

Among the CEA studies, 92% of CHEERS criteria were met on average. The most common missing criteria were reporting the discount rate for cost and outcomes and characterizing heterogeneity (e.g., describing how outcomes may differ between patients with different baseline characteristics). For the disease cost studies, 86% of the criteria (modified from CHEERS checklist) were met on average. Many disease cost studies did not characterize heterogeneity, report methods for how costs were adjusted to the year they were reported, nor provide details on analytical methods (e.g., approach to missing data). Quality assessments are shown in the Additional file 1: Tables S2 and S3.

## Discussion

ILD is associated with substantial direct and indirect costs. The median total direct and indirect costs per year equated to 50% and 17% of GDP per capita, respectively, which is higher than the costs of asthma and COPD [30, 31]. An overall cost could not be determined as the direct and indirect costs were not directly comparable. The search was created to broadly identify economic evaluation in all ILDs; however, most of the literature focused on IPF, highlighting a significant evidence gap on the economic burden of non-IPF ILDs and the cost-effectiveness of therapies beyond antifibrotics. Understanding the costs associated with ILD is an important first step to identify areas for cost mitigation and ways to better support patients.

There was a striking difference in costs among countries. For example, the median annual total direct cost was 66% of the GDP per capita in the US, compared to 6% in Korea. The difference in per capita drug spending is due in part to higher drug costs in the US with high out-of-pocket costs and a large uninsured population. The US also has different policies such as the Orphan Drug Act that provides market exclusivity, allowing drug makers to charge higher costs given the lack of competition [32]. Differences in healthcare systems also contribute to higher costs. In particular, the US has a for-profit insurance system which partly explains why its hospitalization costs are much higher than other countries. Economic evaluations from other countries are greatly needed to better appreciate the global financial burden of ILD. Lastly, costs were impacted by the study time period. The use of antifibrotics for the treatment of IPF became standard of care in 2014 resulting in higher direct costs after this year. Among the 2 IPF studies with study periods after 2014 [33, 34], the mean annual total cost per patient was $83,089 compared to $27,279 before 2014.

The median INB for antifibrotics compared to best supportive care was $88,661, while it was $6536 for nintedanib compared to pirfenidone. Using standard thresholds of cost-effectiveness (e.g., an INB > 0 is considered cost-effective), antifibrotics would not be cost-effective compared to best supportive care, but there may be differences in cost-effectiveness between the available antifibrotics. The only other cost-effective therapy was ambulatory oxygen. Therapies for rare diseases are typically more expensive due to the high expenditures required to show safety and efficacy of therapies using smaller sample sizes. As a result, alternative metrics (e.g., higher willingness-to-pay thresholds) to evaluate cost-effectiveness in orphan diseases should be considered.

There was a paucity of CEAs in non-IPF ILDs, with 6 of 7 studies looking at IPF. Recent data supports the use of antifibrotic medication in non-IPF progressive fibrosing ILD and health agencies have approved its expanded use [35, 36]. With 13–40% of patients with ILD estimated to develop a progressive fibrosing phenotype [37], healthcare spending will certainly increase and economic evaluation for this patient population is a more immediate research priority. Furthermore, there are no economic evaluations on other commonly used ILD therapies such as immunomodulatory medications. In order for CEAs to be conducted, researchers should consider collecting preference-based measures of health-related quality of life (e.g., EQ-5D) as part of their clinical research.

To the best of our knowledge, this is the first systematic review of disease cost and CEA studies in ILD and is an important contribution to health-services management. It provides a comprehensive overview of the magnitude of ILD costs that changed over time, with particular focus on how antifibrotics have impacted costs. We used proportion of GDP per capita to compare costs and cost-effectiveness between countries and identified key limitations and gaps in the evidence base. Critical research priorities for future work include evaluating cost-effectiveness of therapies in non-IPF ILDs and identifying ways to reduce hospitalization rates given it is a leading driver of costs. This study had several limitations. We were unable to conduct a meta-analysis due to the heterogeneity across studies. In addition, the exclusion of non-English studies and conference abstracts could have reduced the number of studies from non-Western countries and, as a result, limit generalizability. Furthermore, we used the $50,000 threshold when calculating the INB to determine cost-effectiveness of therapies. The $50,000 per life year or QALY gained is a commonly used figure of willingness-to-pay threshold in CEA, in particular in the US [38]. We acknowledge that the $50,000 figure is arbitrary, owing more to being a round number than to a well-formulated justification for a specific dollar value. As a result, we conducted a sensitivity analysis replacing the $50,000 threshold with 1-time and 3-times the GDP per capita which are frequently used as willingness-to-pay thresholds in global health [39]. The 1-time GDP per capita threshold can scale the values of a life year to the resources available in each country, while the 3-times GDP per capita reflects the scenario where value per life year is greater than the GDP per capita. In our study, the therapies remained cost-effective when the different thresholds were employed.

## Conclusions

The direct and indirect costs associated with ILD are consistently high in all countries with available data, with the cost-effectiveness profiles of new therapies generally undesirable. The global median total direct cost equates to 50% of a country’s GDP per capita and has been increasing over time. Cost data on non-IPF ILD and therapies beyond antifibrotics should be research priorities.

## Supplementary Information

**Additional file 1.** Search strategy for MEDLINE and Embase using the Ovid platform. **Table S1**. Sensitivity analysis for cost-effectiveness analyses. **Table S2**. Risk of bias assessment for disease cost studies. **Table S3**. Risk of bias assessment for cost-effectiveness analyses. **Table S4**. Study characteristics.

## Data Availability

The datasets generated during and/or analysed during the current study are available from the corresponding author on reasonable request.

## Abbreviations

CEA: Cost-effectiveness analysis
EQ-5D: EuroQoL 5 dimensions
GDP: Gross domestic product
ICER: Incremental cost-effectiveness ratio
ILD: Interstitial lung disease
INB: Incremental net benefit
IPF: Idiopathic pulmonary fibrosis
QALY: Quality adjusted life year
