# Supplementary material for: A systematic review on the economic burden of interstitial lung disease and the cost-effectiveness of current therapies
Source: BMC Pulm Med. 2022 Apr 20;22:148. doi: 10.1186/s12890-022-01922-2 (PMC9020025; doi:10.1186/s12890-022-01922-2)
Supplement: Supplementary file 1 — Additional file 1. Search strategy for MEDLINE and Embase using the Ovid platform. Table S1. Sensitivity analysis for cost-effectiveness analyses. Table S2. Risk of bias assessment for disease cost studies. Table S3. Risk of bias assessment for cost-effectiveness analyses. Table S4. Study characteristics. [file 12890_2022_1922_MOESM1_ESM.docx]

**SUPPLEMENT**

**Search strategy for MEDLINE and Embase using the Ovid platform.** The search was developed in consultation with an academic librarian.

1. exp Lung Diseases, Interstitial/

2. exp Pulmonary Fibrosis/

3. (interstitial adj4 lung).mp.

4. diffuse parenchymal lung disease*.mp.

5. (interstitial adj4 pneumon*).mp.

6. (pulmonary adj4 fibrosis).mp.

7. (fibrosis adj4 lung*).mp.

8. (scarring adj4 lung*).mp.

9. granulomatos*.mp.

10. silicosis.mp.

11. asbestosis.mp.

12. hypersensitivity pneumon*.mp.

13. berylliosis.mp.

14. alveolitis.mp.

15. or/1-14

16. *"health care economics and organizations"/ or exp economics/

17. (cost* adj3 (burden* or direct* or indirect* or medical or "non medical" or "health care" or healthcare or hospital or health or econom* or societ* or product*)).mp.

18. (econ* adj3 (burden* or direct* or indirect* or medical or "non medical" or "health care" or healthcare or hospital or health or societ* or product*)).mp.

19. (expense* adj3 (burden* or direct* or indirect* or medical or "non medical" or "health care" or healthcare or hospital or health or econom* or societ* or product*)).mp.

20. (financ* adj3 (burden* or direct* or indirect* or medical or "non medical" or "health care" or healthcare or hospital or health or econom* or societ* or product*)).mp.

21. or/16-20

22. 15 and 21

**Table S1. Sensitivity analysis for cost-effectiveness analyses.** The incremental net benefit (INB) was calculated using a standard willingness-to-pay threshold of $50,000 USD. A sensitivity analysis using different thresholds (1-time and 3-times the country’s GDP) was conducted to evaluate whether treatments remained cost-effective at different thresholds that were more reflective of a country’s willingness-to-pay. INB values > 0 represent cost-effective interventions (bold text). Costs are shown in 2020 USD. *Cost and KBILD values for each of the treatment and control groups were not available. The incremental cost and KBILD score are shown, with the ICER representing the cost per unit increase in KBILD. *Abbreviations:* KBILD, King’s Brief Interstitial Lung Disease; M, million; MS, microsimulation; PSS, personal social services; Tx, treatment; UK NHS, United Kingdom National Health Service.

| **First author**  **Study year** | **Country** | **ILD** | **Treatment (tx)** | **Tx cost** | **Tx QALY / person** | **Control** | **Control cost** | **Control QALY / person** | **GDP per capita (2020 USD)** | **INB ($50,000)** | **INB**  **(1 x GDP)** | **INB**  **(3 x GDP)** |
| --- | --- | --- | --- | --- | --- | --- | --- | --- | --- | --- | --- | --- |
| **Clay [1]**  2019 | France | IPF | Nintedanib | 140,486 | 4.5 | **Pirfenidone** | 133,592 | 5.2 | 40,878 | -41,894 | -35,509 | -92,738 |
| **Porte [2]**  2018 | France | IPF | **Nintedanib** | 109,100 | 3.34 | Pirfenidone | 118,025 | 3.29 | 42,822 | 11,425 | 11,066 | 15,348 |
| **Rinciog [3]**  2017 | UK | IPF | **Nintedanib** | 148,950 | 3.5 | Pirfenidone | 152,986 | 3.45 | 42,557 | 6,536 | 6,164 | 10,420 |
| **Loveman [4]**  2015 | UK | IPF | Nintedanib | 272,273 | 4.01 | **Pirfenidone** | 128,523 | 3.34 | 49,180 | -110,250 | -110,799 | -44,898 |
| **Rinciog [5]**  2020 | Belgium | IPF | **Nintedanib** | 138,191 | 3.36 | Pirfenidone | 151,705 | 3.28 | 44,594 | 17,514 | 17,082 | 24,217 |
| **Clay [1]**  2019 | France | IPF | Nintedanib | 140,486 | 4.5 | **BSC** | 19,039 | 3.8 | 40,878 | -86,447 | -92,832 | -35,603 |
| **Porte [2]**  2018 | France | IPF | Nintedanib | 109,100 | 3.34 | **BSC** | 22,056 | 2.98 | 42,822 | -69,044 | -71,628 | -40,796 |
| **Rinciog [3]**  2017 | UK | IPF | Nintedanib | 148,950 | 3.5 | **BSC** | 38,076 | 3.1 | 42,557 | -90,874 | -93,851 | -59,806 |
| **Loveman [4]**  2015 | UK | IPF | Nintedanib | 272,273 | 4.01 | **BSC** | 6,014 | 2.98 | 49,180 | -214,759 | -215,604 | -114,293 |
| **Clay [1]**  2019 | France | IPF | Pirfenidone | 133,592 | 5.2 | **BSC** | 19,039 | 3.8 | 40,878 | -44,553 | -57,324 | 57,135 |
| **Porte [2]**  2018 | France | IPF | Pirfenidone | 118,025 | 3.29 | **BSC** | 22,056 | 2.98 | 42,822 | -80,469 | -82,694 | -56,145 |
| **Rinciog [3]**  2017 | UK | IPF | Pirfenidone | 152,986 | 3.45 | **BSC** | 38,076 | 3.1 | 42,557 | -97,410 | -100,015 | -70,225 |
| **Loveman [4]**  2015 | UK | IPF | Pirfenidone | 128,523 | 3.34 | **BSC** | 6,014 | 2.98 | 49,180 | -104,509 | -104,804 | -69,395 |
| **Whitty [6]**  2019 | UK | IPF | **Ambulatory oxygen** | 128 | 3.7 | No oxygen | - | - | 42,877 | 184,872 | 158,517 | 475,807 |
| **Groen [7]**  2004 | Netherlands | Any ILD | Lung transplantation | 138M | 1203 | **No lung transplant** | 71.25M | 738 | 55,403 | -17M | 26M | 77M |

**Table S2.** **Risk of bias assessment for disease cost studies.** The CHEERS criteria that were applicable to disease cost studies were used to assess risk of bias with a maximum score of 13 for studies that had a comparator or 12 for studies without comparators. The criteria that were not met corresponds to the item number in the CHEERS checklist.

| **Study** | **Score** | **Maximum score** | **% of criteria met** | **Criteria not met** |
| --- | --- | --- | --- | --- |
| Collard [1] | 12 | 13 | 92 | 17 |
| Collard [2] | 12 | 13 | 92 | 17 |
| Mortimer [3] | 10 | 12 | 83 | 14, 17 |
| Raimundo [4] | 9 | 12 | 75 | 17, 21, 24 |
| Corral [5] | 12 | 13 | 92 | 17 |
| Kalluri [6] | 11 | 13 | 85 | 17, 23 |
| Tarride [7] | 11 | 12 | 92 | 21 |
| Kim [8] | 9 | 12 | 75 | 14, 17, 21 |
| Hilberg [9] | 9 | 13 | 69 | 2, 17, 21, 22 |
| Olson [10] | 10 | 13 | 77 | 14, 17, 21 |
| Frank [11] | 10 | 12 | 83 | 14, 21 |
| Morrisroe [12] | 12 | 13 | 92 | 14 |
| Gayle [13] | 11 | 12 | 92 | 17 |
| Fisher [14] | 13 | 13 | 100 |  |
| Zhou [15] | 12 | 13 | 92 | 21 |
| Raimundo [16] | 9 | 12 | 75 | 17, 21, 24 |
| Algamdi [17] | 12 | 12 | 100 |  |
| Algamdi [18] | 12 | 12 | 100 |  |
| Yu [19] | 10 | 12 | 83 | 17, 21 |
| Mooney [20] | 11 | 12 | 92 | 21 |
| Fan [21] | 12 | 12 | 100 |  |
| Cottin [22] | 10 | 12 | 83 | 17, 21 |
| Navaratnam [23] | 11 | 12 | 92 | 21 |
| Pedraza-serra [24] | 9 | 12 | 75 | 14, 17, 21 |
| Hernandez-Gomez [25] | 7 | 12 | 58 | 14, 17, 21, 23, 24 |

**Table S3. Risk of bias assessment for cost-effectiveness analyses.** Studies were evaluated using the Consolidated Health Economic Evaluation Reporting Standards (CHEERS), which has a maximum total score of 24.

| **Study** | **Score** | **Maximum score** | **% of CHEERS criteria met** | **CHEERS criteria not met** |
| --- | --- | --- | --- | --- |
| Clay [26] | 22 | 24 | 92 | 9, 21 |
| Porte [27] | 23 | 24 | 96 | 21 |
| Rinciog [28] | 23 | 24 | 96 | 21 |
| Rinciog [29] | 23 | 24 | 96 | 21 |
| Loveman [30] | 23 | 24 | 96 | 21 |
| Whitty [31] | 21 | 24 | 88 | 9, 15, 16 |
| Groen [32] | 20 | 24 | 83 | 6, 21, 23, 24 |

**Table S4. Study characteristics.** *Range of median age at time of death across study subgroups. Age is shown as mean±standard or median (interquartile range). Other races include Asians, North American Native, and others. *Abbreviations:* CTD-ILD, connective tissue disease-associated interstitial lung disease; IIP, idiopathic interstitial pneumonia; IPF, idiopathic pulmonary fibrosis; NA, not available; PF, progressive fibrosing; RA, rheumatoid arthritis; SSc, systemic sclerosis.

| **Study** | **ILD type** | **Method of ILD diagnosis** | **Country** | **Time period** | **Sample size** | **Age** | **Males (%)** | **Ever smoker (%)** | **Race or Ethnicity (%)** |
| --- | --- | --- | --- | --- | --- | --- | --- | --- | --- |
| **Direct costs** | | | | | | | | | |
| Collard [1] | IPF | ICD codes | US | 2001-2008 | 9286 | 74 ± 9.2 | 55 | - | - |
| Collard [2] | IPF | ICD codes | US | 2000-2011 | 7855 | 79±7 | 43 | - | White 92, Black 4, Hispanic 2, Other 2 |
| Mortimer [3] | IPF | ICD codes | US | 2008-2014 | 4716 | 77±6 | 50 | - | White 74, Black 10, Hispanic 6, Other 10 |
| Raimundo [4] | IPF | ICD codes | US | 2009-2011 | 3619 | 74±NA | 49 | - | - |
| Corral [5] | IPF | ICD codes | US | 2014-2018 | 1455 | 71±10 | 69 | - | - |
| Kalluri [6] | IPF | Patient cohort | Canada | 2012-2018 | 2768 | 73-86* | 57 | - | - |
| Tarride [7] | IPF | ICD codes | Canada | 2006-2011 | 5152 | 77±12 | 57 | - | - |
| Kim [8] | IPF | ICD codes | Korea | 2009-2013 | 18,006 | 67±13 | 63 | - | - |
| Hilberg [9] | IPF | Patient cohort | Denmark | 2003-2009 | 120 | 67±NA | 77 | - | - |
| Olson [10] | Non-IPF  PF-ILD | ICD codes | US | 2014-2016 | 373 | - | - | - | - |
| Frank [11] | IIP (I) | ICD codes | Germany | 2010-2013 | 14,453 | 72±11 | 60 | - | - |
| Morrisroe [12] | SSc-ILD | Patient cohort | Australia | 2008-2015 | 335 | 55±13 | 20 | 27 | White 88, Asian 11 |
| Gayle [13] | SSc-ILD | ICD codes | England | 2005-2016 | 127 | 71±13 | 25 | 33 | White 82, Black 4, Other 14 |
| Fisher [14] | SSc-ILD | ICD codes | US | 2003-2014 | 219 | 57±13 | 19 | - | - |
| Zhou [15] | SSc-ILD | ICD codes | US | 2004-2016 | 52 | 60±13 | 19 | - | - |
| Raimundo [16] | RA-ILD | ICD codes | US | 2004-2013 | 11,845 | 65±NA | 31 | 5 | - |
| **Indirect costs** | | | | | | | | | |
| Algamdi [17] | Fibrotic ILD | Patient cohort | Canada | 2015-2017 | 148 | 61±10.1 | 61 | 62 | - |
| Algamdi [18] | CTD-ILD | Patient cohort | Canada | 2015-2017 | 113 | 53±11 | 37 | 44 | - |
| Zhou [15] | SSc-ILD | ICD codes | US | 2004-2016 | 52 | 60±13 | 19 | - | - |
| Hilberg [9] | IPF | Patient cohort | Denmark | 2003-2009 | 120 | 67±NA | 77 | - | - |
| **Specific direct costs** | | | | | | | | | |
| Yu [19] | IPF | ICD codes | US | 2006-2011 | 1735 | 72±13 | 54 | - | - |
| Mooney [20] | IPF | ICD codes | US | 2009-2011 | 22,350 | 70±0.3 | 49 | - | White 64, Black 8, Hispanic 9, Other 19 |
| Fan [21] | IPF | Patient cohort | US | 2014-2016 | 300 | 69±8 | 75 | 68 | White 95, Black 1, Other 4 |
| Cottin [22] | IPF | ICD codes | France | 2008-2013 | 6476 | 77 (69-83) | 56 | - | - |
| Navaratnam [23] | IPF | ICD codes | England | 1998-2010 | 48,415 | 68±NA | 54 | - | - |
| Pedraza-serra [24] | IPF | ICD codes | Spain | 2004-2013 | 12,739 | 73±12 | 57 | - | - |
| Hernandez-Gomez [25] | Fibrotic ILD | Patient cohort | Spain | 2011-2014 | 33 | 64 (30-79) | 33 | - | - |

**References**

1. Collard HR, Ward AJ, Lanes S*, et al.* Burden of illness in idiopathic pulmonary fibrosis. *J Med Econ* 2012: 15(5): 829-835.

2. Collard HR, Chen S-Y, Yeh W-S*, et al.* Health Care Utilization and Costs of Idiopathic Pulmonary Fibrosis in US Medicare Beneficiaries Aged 65 Years and Older. *Annals of the American Thoracic Society* 2015: 12(7): 981-987.

3. Mortimer K, Hartmann N, Chan C*, et al.* Characterizing idiopathic pulmonary fibrosis patients using US Medicare-advantage health plan claims data. *BMC Pulmonary Medicine* 2019: 19(1): 11.

4. Raimundo K, Solomon JJ, Olson AL*, et al.* Rheumatoid Arthritis-Interstitial Lung Disease in the United States: Prevalence, Incidence, and Healthcare Costs and Mortality. *J Rheumatol* 2019: 46(4): 360-369.

5. Corral M, DeYoung K, Kong AM. Treatment patterns, healthcare resource utilization, and costs among patients with idiopathic pulmonary fibrosis treated with antifibrotic medications in US-based commercial and Medicare Supplemental claims databases: a retrospective cohort study. *BMC Pulm Med* 2020: 20(1): 188.

6. Kalluri M, Lu-Song J, Younus S*, et al.* Health Care Costs at the End of Life for Patients with Idiopathic Pulmonary Fibrosis. Evaluation of a Pilot Multidisciplinary Collaborative Interstitial Lung Disease Clinic. *Ann Am Thorac Soc* 2020: 17(6): 706-713.

7. Tarride JE, Hopkins RB, Burke N*, et al.* Clinical and economic burden of idiopathic pulmonary fibrosis in Quebec, Canada. *Clinicoecon Outcomes Res* 2018: 10: 127-137.

8. Kim SW, Myong JP, Yoon HK*, et al.* Health care burden and medical resource utilisation of idiopathic pulmonary fibrosis in Korea. *Int J Tuberc Lung Dis* 2017: 21(2): 230-235.

9. Hilberg O, Bendstrup E, Ibsen R*, et al.* Economic consequences of idiopathic pulmonary fibrosis in Denmark. *ERJ Open Res* 2018: 4(2).

10. Olson AL, Swigris JJ. Idiopathic pulmonary fibrosis: diagnosis and epidemiology. *Clin Chest Med* 2012: 33(1): 41-50.

11. Frank AL, Kreuter M, Schwarzkopf L. Economic burden of incident interstitial lung disease (ILD) and the impact of comorbidity on costs of care. *Respir Med* 2019: 152: 25-31.

12. Morrisroe K, Stevens W, Sahhar J*, et al.* The clinical and economic burden of systemic sclerosis related interstitial lung disease. *Rheumatology (Oxford)* 2020: 59(8): 1878-1888.

13. Gayle A, Schoof N, Alves M*, et al.* Healthcare Resource Utilization Among Patients in England with Systemic Sclerosis-Associated Interstitial Lung Disease: A Retrospective Database Analysis. *Adv Ther* 2020: 37(5): 2460-2476.

14. Fischer A, Kong AM, Swigris JJ*, et al.* All-cause Healthcare Costs and Mortality in Patients with Systemic Sclerosis with Lung Involvement. *J Rheumatol* 2018: 45(2): 235-241.

15. Zhou Z, Fan Y, Thomason D*, et al.* Economic Burden of Illness Among Commercially Insured Patients with Systemic Sclerosis with Interstitial Lung Disease in the USA: A Claims Data Analysis. *Advances in Therapy* 2019: 36(5): 1100-1113.

16. Raimundo K, Chang E, Broder MS*, et al.* Clinical and economic burden of idiopathic pulmonary fibrosis: a retrospective cohort study. *BMC Pulm Med* 2016: 16: 2.

17. Algamdi M, Sadatsafavi M, Fisher JH*, et al.* Costs of Workplace Productivity Loss in Patients With Fibrotic Interstitial Lung Disease. *Chest* 2019: 156(5): 887-895.

18. Algamdi M, Sadatsafavi M, Fisher JH*, et al.* Costs of Workplace Productivity Loss in Patients with Connective Tissue Disease-associated Interstitial Lung Disease. *Ann Am Thorac Soc* 2020: 17(9): 1077-1084.

19. Yu YF, Wu N, Chuang C-C*, et al.* Patterns and Economic Burden of Hospitalizations and Exacerbations Among Patients Diagnosed with Idiopathic Pulmonary Fibrosis. *Journal of Managed Care & Specialty Pharmacy* 2016: 22(4): 414-423.

20. Mooney JJ, Raimundo K, Chang E*, et al.* Hospital cost and length of stay in idiopathic pulmonary fibrosis. *J Med Econ* 2017: 20(5): 518-524.

21. Fan Y, Bender SD, Conoscenti CS*, et al.* Hospital-Based Resource Use and Costs Among Patients With Idiopathic Pulmonary Fibrosis Enrolled in the Idiopathic Pulmonary Fibrosis Prospective Outcomes (IPF-PRO) Registry. *Chest* 2020: 157(6): 1522-1530.

22. Cottin V, Schmidt A, Catella L*, et al.* Burden of Idiopathic Pulmonary Fibrosis Progression: A 5-Year Longitudinal Follow-Up Study. *PLoS One* 2017: 12(1): e0166462.

23. Navaratnam V, Fogarty AW, Glendening R*, et al.* The increasing secondary care burden of idiopathic pulmonary fibrosis: hospital admission trends in England from 1998 to 2010. *Chest* 2013: 143(4): 1078-1084.

24. Pedraza-Serrano F, Lopez de Andres A, Jimenez-Garcia R*, et al.* Retrospective observational study of trends in hospital admissions for idiopathic pulmonary fibrosis in Spain (2004-2013) using administrative data. *Bmj Open* 2017: 7(2): -e013156.

25. Hernandez-Gonzalez F, Lucena CM, Ramirez J*, et al.* Cryobiopsy in the diagnosis of diffuse interstitial lung disease: yield and cost-effectiveness analysis. *Arch Bronconeumol* 2015: 51(6): 261-267.

26. Clay E, Cristeau O, Chafaie R*, et al.* Cost-effectiveness of pirfenidone compared to all available strategies for the treatment of idiopathic pulmonary fibrosis in France. *Journal of Market Access & Health Policy* 2019: 7(1): 1626171.

27. Porte F, Cottin V, Catella L*, et al.* Health economic evaluation in idiopathic pulmonary fibrosis in France. *Curr Med Res Opin* 2018: 34(10): 1731-1740.

28. Rinciog C, Watkins M, Chang S*, et al.* A Cost-Effectiveness Analysis of Nintedanib in Idiopathic Pulmonary Fibrosis in the UK. *Pharmacoeconomics* 2017: 35(4): 479-491.

29. Rinciog C, Diamantopoulos A, Gentilini A*, et al.* Cost-Effectiveness Analysis of Nintedanib Versus Pirfenidone in Idiopathic Pulmonary Fibrosis in Belgium. *Pharmacoecon Open* 2020: 4(3): 449-458.

30. Loveman E, Copley VR, Colquitt J*, et al.* The clinical effectiveness and cost-effectiveness of treatments for idiopathic pulmonary fibrosis: a systematic review and economic evaluation. *Health Technol Assess* 2015: 19(20): i-xxiv, 1-336.

31. Whitty JA, Rankin J, Visca D*, et al.* Cost-effectiveness of ambulatory oxygen in improving quality of life in fibrotic lung disease: preliminary evidence from the AmbOx Trial. *Eur Respir J* 2020: 55(2).

32. Groen H, van der Bij W, Koeter GH*, et al.* Cost-effectiveness of lung transplantation in relation to type of end-stage pulmonary disease. *Am J Transplant* 2004: 4(7): 1155-1162.
